# Gifted Children, Youth Culture, and Popular Individualism in 1970s and 1980s Britain

**DOI:** 10.1017/S0018246X21000844

**Published:** 2022-03-23

**Authors:** Jennifer Crane

**Affiliations:** Faculty of History, University of Oxford, Oxford, UK

## Abstract

This article examines a niche space: youth clubs created by small voluntary organizations for ‘gifted children’ in 1970s and 1980s Britain. It asks how individualism shaped everyday life and demonstrates how youth culture was much broader than just the permissiveness that dominates the literature. Gifted children are currently missing from accounts of modern Britain, which focus on mainstream educational categories. Yet, including them in our analysis provides new insights into the diversity of youth cultures that existed in these decades. Drawing on new uncatalogued archives, and centrally poetry, letters, and stories from young people themselves, the article shows that conservative and radical visions co-existed. Young people shaped their own culture, subverting and challenging ideas of themselves as distinctive future leaders.

## I

‘I feel that seeing myself as an individual is something I have to do for myself… Some day I will find myself – soon, I hope – and be able to express myself to others, but I need you, as well as other people, to help me.’^1^ This letter was written collectively by fourteen- to sixteen-year-olds enrolled on a ‘Mentally Gifted Programme’ in Alhambra, California. It was published in a newsletter designed for British members of the National Association for Gifted Children in 1979. The young authors emphasized that they felt like distinctive individuals, because they were intellectually ‘gifted’. As such, they needed a non-specified ‘you’ and also ‘other people’ to help them. While vaguely constructed, the ideas that ‘gifted child’ was a meaningful cultural and social identity, and that ‘gifted children’ needed distinct spaces, were well understood by British peers; the editor of the National Association’s newsletter wrote that, ‘judging by the letters I’ve had in’, this was a topic child-members ‘feel very strongly about’.^2^ This article uses such letters from the gifted young, alongside their poetry and essays, and those from adult voluntary leaders, to thoroughly excavate the distinct spaces created for young people labelled as ‘intellectually gifted’ in 1970s and 1980s Britain. In doing so, it shows how diverse ‘youth culture’ was in this period, looking beyond the trends of the ‘permissive sixties’. Indeed, the article argues that a more useful lens through which to understand these spaces is popular individualism: while the post-war welfare state had never catered for the gifted young, it was only in the 1970s and 1980s that parents took action, dedicating significant time, effort, and emotional resources to change this. Examining the resulting small, localized spaces of gifted youth culture shows the extent to which ideas of ‘the individual’ reshaped lives in 1970s and 1980s Britain, and the variety of ‘youth cultures’ in these decades, critically shaped by the young themselves.

The term ‘gifted child’ itself, like many new labels and identities, proliferated in use from the 1970s and 1980s. This term first originated in the nineteenth century, but the idea gained scientific authority when new psychometric tests of the early twentieth century, primarily developed in Britain, Europe, and America, developed ‘measures’ of giftedness.^3^ While there were a broad variety of such tests, typically to qualify as ‘gifted’ a child had to perform exceptionally highly: on an IQ test, for example, designed to have an average score of 100, to be ‘gifted’ a child would have to score 120 or, by some measures, perhaps even 140. While psychometric testing became influential across the Global North – for example in school admissions, job recruitment, and military qualifications – American press, policy, and parents paid the most attention to identifying gifted children. Amidst the Cold War, American films and novels provided popular fictional accounts of the supernatural or mystical origins of these young people.^4^ National and federal governments also gave significant funding to schools looking to help the gifted, hoping to furnish scientific advantage – this resulted in a proliferation of special schools for the gifted, special streams within existing schools, and extra-curricula provision.^5^

In Britain, by contrast, there was very little educational or parental provision for children labelled as ‘intellectually gifted’ in the twentieth century; the National Association, which this article focuses on, was unusual. Britain’s post-war welfare state aimed to cater for children of all ‘aptitude and ability’. The most academically focused form of education available in this system – the grammar school – educated between 24.6 per cent and 37.8 per cent of the secondary school-aged population between the late 1940s and start of the 1960s.^6^ The gifted young did not receive specific sub-provision within this system, and this was not typically a category in political or press debate. When occasionally raised in parliament or local press by interested politicians or teachers, indeed, popular responses emphasized that provision for this subgroup must be rejected as ‘elitist’.^7^ Despite small, local, educational experiments in extra enrichment provision for ‘the gifted’, for example in Essex, Oxford, and Devon, by 1977 a report by Her Majesty’s Inspectorate still stated that: ‘For the vast majority of schools and their teachers’, gifted education was ‘neither implicit nor explicit in the day-to-day dialogue of school life’.^8^ This changed significantly at the turn of the twenty-first century: the New Labour governments sought to identity gifted youth, ordering schools to provide enrichment classes and to keep a list of the gifted, and also partially funding a National Academy for Gifted Youth, to run extra-curricula courses.^9^ Overall, nonetheless, the gifted child was not a strong feature of twentieth-century British policy: reflecting this, this category is not discussed in recent large-scale accounts of the welfare state or history of education.^10^

Despite this, this article argues that studying the small and highly distinctive youth cultures that were established for, and lived by, gifted young people can be hugely significant for modern British historians. This article accesses these youth cultures through analysis of the National Association for Gifted Children. A small but fascinating group, this was founded in 1966 by Margaret Branch, a dynamic psychiatric social worker whose interest in this area was sparked by working with one ‘disturbed and epileptic boy with an IQ of 150’.^11^ Initially, Branch focused on campaigning to raise awareness of this cause, and she gained a small amount of broadsheet newspaper coverage in the 1970s, arguing that providing for the gifted was an equalities issue, and not elitist at all.^12^ By the 1980s, the National Association became more popular – it had 4,863 members by 1981 – but it also became more decentralized, as interested parents had joined from across the United Kingdom and formed fifty regional branches.^13^ Many branches were focused on service-delivery rather than campaigning, and parent-volunteers organized weekend and holiday clubs for the gifted young. A national central hub remained significant, producing policy reports and newsletters for parents, children, and interested professionals, as well as counselling services. These rich materials – left uncatalogued in boxes at the organization’s successor – are the primary source-base for this article.

Analysing the youth spaces created by the National Association, this article brings significant new ways of thinking to bear on our understandings of youth culture, the permissive society, and popular individualism. The constellation of activities organized for the gifted young – around nature, woodwork, classical and imperial history, and scientific innovation, for example – show the limitations of dominant historiographical and sociological accounts of youth culture in the 1960s and 1970s, which emphasize a framework of permissiveness: that youth culture meant the development of new consumer and sexual behaviours, and subversive or counter-cultural trends in fashion, music, and leisure.^14^ These ideas have been nuanced by sociological researchers in the 1960s, such as Mark Abrams and Pearl Jephcott, and by historians writing in recent years, such as Helena Mills and Jim Gledhill, who have suggested that the majority of youth lives were not engaged in these trends, but rather ‘mundane’ or ‘ordinary’.^15^ Yet, this article’s case-study can significantly extend this analysis, and show what, exactly, youth culture meant for one group, who felt far from ‘ordinary’ but were also not ‘permissive’ – the gifted.

Beyond showing the variation in ‘youth cultures’, this article also provides a case-study of how individualism was lived in daily life. The article argues that gifted youth culture was not intended as a ‘vanguard’ against the ‘perceived threat of “modernity”’, to draw on Sian Edwards’s characterization of the work of the Young Farmers’ Club decades earlier.^16^ Rather, adult-organizers invited the gifted young to see themselves as a new social group – thinkers and subjects significantly defined by their intellect and who, as a result, would want to socialize together, and potentially rise as ‘future leaders’. Existing historiographical accounts typically focus on individualism as a political phenomenon, looking at when this idea emerged in political rhetoric, how it became established, and how it shaped electoral and consumer behaviours.^17^ This article, however, complements newer works, for example by Emily Robinson, Camilla Schofield, Florence Sutcliffe-Braithwaite, Natalie Thomlinson, and Jon Lawrence, which have started to offer rich smaller case-studies about how individualism was lived, extending analysis of this concept beyond policy-making into daily life, and beyond ‘Thatcherism’ into surrounding decades.^18^ Notably, this article offers a small case-study that, precisely because of its niche nature, demonstrates how far-flung, varied, and powerful ideas of individualism were in shaping distinct subcultural social activity in daily life in the 1970s and 1980s. Specifically, the article shows that parents organizing gifted groups felt that their distinct experiences and emotions were not catered for in the welfare state. Their response was not a turn towards isolation, nor to lobby for state provision, but rather to forge new communities through collective action, becoming providers themselves. Long-standing ideas of community then remained present in this activism, and yet it was also motivated by individualist ideas about entitlement and new expressions of emotion.

A final contribution of this article is its insistence that historical scholarship must take more seriously the ways in which young people themselves have structured social, cultural, and political life. The article uses a methodological framework of agency, drawing on recent works in sociology and history.^19^ These theoretical literatures have demonstrated that we must not simply try to ‘find’ agency in history, nor, as Chris Millard warns, valorize any evidence of ‘experience’.^20^ Rather, we must assume that young people had a level of power in any encounters in the social world, but that this power will have been exercised relationally, dependent on environment and the young person’s own identity. This is not always recognized in histories of youth culture. Heather Shore’s review of David Fowler’s influential work in youth culture, indeed, noted that this historiography rarely considers ‘the agency of the young in the making of their own culture’.^21^ More broadly, recent historiographical reviews by Colin Heywood, Harry Hendricks, and Sarah Maza have argued that ‘age struggles to assert itself as a category of analysis beside class, gender, and ethnicity in historical study’, because of a perceived lack of sources and because children are not ‘an organized political constituency’.^22^ This article challenges these assumptions. It shows the value of child-produced sources, demonstrating that these are available particularly in uncatalogued materials, looking beyond existing archives.^23^ The article demonstrates, also, that young people may not have organized as a political constituency, but that they did exert power: changing the views and leisure activities of their parents, voluntary leaders, and their teachers and peers at school, as they transferred ideas between ‘gifted’ and ‘non-gifted’ spaces.

Structurally, this article first discusses what we can know about the demographics of the National Association, and the motivations of its involved parents and children. It demonstrates the many gaps in informal, uncatalogued archives, but also maps out the likely biases involved in identifying ‘the gifted’. This section shows also how parents’ activism was driven by a distinct sense that collective action was the appropriate response to their individual experiences and emotions. Second, the article examines the distinct model of gifted youth culture lived in the Association’s youth activities, as planned by adults, showing their distinctive, ‘individual’ nature. Third, the article explores how young people received and reshaped these spaces; this demonstrates the power of the young themselves in shaping ‘youth culture’. This seemingly small case-study, then, can demonstrate the pervasiveness and meanings of individualism in 1970s and 1980s Britain, and its implications for our thinking about youth cultures.

## II

We cannot perfectly trace the demographics of those who attended and organized the National Association’s fifty regional clubs in the 1970s and 1980s, due to the fractured nature of available archival evidence about this group. However, we can understand the Association’s efforts to recruit from minoritized populations, and the challenges it faced in doing so. From parental writings, we can also understand the motivations of the Association’s voluntary organizers.

The National Association did not require a specific level of ‘IQ’ scoring or other such performance on psychometric testing to invite young people to join its clubs. A publication by the Association from 1989, *Bright children*, stated that intelligence tests ‘can be useful’, but were ‘not particularly good predictors’ nor ‘very accurate measures of intellectual ability’.^24^ This observation was supported by contemporary sociological research in the 1980s and 1990s, particularly from America, which emphasized that psychometric testing was significantly biased towards white, middle-class boys.^25^ Indeed, recent historical research by Rob Waters has shown how this affected the education of Black children, who were disproportionately sent to special needs schools on the basis of such tests.^26^ In the absence of an effective metric, and also looking to help any parent who believed they needed support, the Association allowed families to self-identify their children as gifted and to sign up. Parents were charged a fee that organizers sought to keep low: in 1971, £2 per year ‘for a husband and wife’, 50p for students who were training, and £4 for ‘schools and other educational establishments’.^27^ While typically referred by parents, some young people were recommended to join by local educational psychologists or teachers, particularly when partnerships between local Association leaders and local education authorities were strong.^28^

Unfortunately, we do not have precise demographic data available, in the uncatalogued Association archives or in supplementary policy and educational documents, about the children or parents who joined the National Association. In part, this reflected conscious decisions of National Association leaders not to question their members about demographics. Earlier organizers of the Association – notably Branch – were passionate about the privacy of the young people involved, and did not wish to collect or to publish their demographic details or indeed photographs.^29^ Augmenting this archival gap, the editor of the Association’s magazines for children did not ask about the class, race, or ethnic backgrounds of children who wrote in, nor about which parts of the country they came from, and she recalled in 2021 that the young people did not mention this.^30^ Trying to read for clues in the surviving publications themselves, names we read as representing ‘boys’ and ‘girls’ are featured equally. Surnames that we may read as more likely to be from first- or second-generation immigrant communities occur only rarely; notably traditionally Chinese surnames featured from the 1980s.

Another factor that makes it difficult to understand the demographic mix of this organization is the variety between the regional clubs, which led on organizing the extra-curricula activities. By November 1980, the organization had established forty-eight clubs, with two more in formation. The majority were in the south-east of England; however, there was also one club in Northern Ireland (in Belfast), two in Scotland (in Strathclyde and the Lothians), and two in Wales (entitled ‘North Wales’ and ‘South Wales’), as well as clubs located across England and one in the Isle of Wight.^31^ Each of these clubs were organized by different volunteers, who, without significant oversight from the National Association, brought their own assumptions, and also catered to distinct regional and local populations and interests. Surviving branch accounts show that branch activities typically attracted regular audiences of between twenty and eighty children, depending on area.^32^ Some groups had support from local education authorities – providing funding, staff, or space, but many did not.^33^ Some were able to raise money from local parents to employ helpers and specialists to assist with running their courses, but many were not.^34^ The majority were organized by parents, but some – such as the Isle of Wight branch – included ‘a primary school headteacher, a middle school headteacher, the principal careers officer and the senior educational psychologist’, interested for professional reasons, on their committees.^35^

We have then only fragmentary evidence through which to understand the social mix of the National Association. We do know, however, that central organizers spoke and wrote a lot about class at the Association, and spoke about the need to include working-class children in their voluntary activities. Yet, we can also question how much these concerns translated to working-class participation – and indeed how inviting statements were, for example, from early Association leaders telling national press that working-class parents must become involved because otherwise their ‘poor home backgrounds’ may ‘suppress’ the chances of their children, leaving them in ‘dead end’ manual jobs.^36^ Comments by the founder of the Lichfield branch, launching this in 1975, that this was a ‘classless organisation’, suggest perhaps a lack of attention here to how to actively facilitate diverse participation.^37^

Yet, we do also know from Association newsletters that by the late 1970s, concerns about class manifested in practical efforts to recruit young people from the working classes and from minoritized ethnic backgrounds. This was a key concern of specific branches, notably in Wolverhampton, the Wirral, Merseyside, and Liverpool, where local organizers were able to start partnerships with local educational authorities also concerned about the educational achievements of deprived and immigrant children.^38^ The local education authority in Liverpool started multiple projects in the inner city in the 1970s and 1980s, looking to overcome childhood underachievement linked to families’ material challenges: ‘poor and overcrowded accommodation; a lack of privacy with high noise levels; large families; many children ill-clothed, ill-slept, ill-fed and ill-medicated; much absence from school; outlooks affected by drab surroundings’ and poverty.^39^ One initiative supported by Liverpool’s local education authority was the establishment of a National Association club in 1978 – the authority provided space in a local comprehensive school, and contacts for the National Association’s local organizers to work with headteachers and staff at local schools to identify potentially gifted young people.^40^ In its first year, the club had 30 members; in its second year, 170 members; a membership that remained steady by the organization’s third year. Child attendees were described in Association newsletters as ‘multi-ethnic’ and ‘multi-racial’; they were mostly boys (102 boys and 76 girls in November 1980) and ranged in age between 2.5 and 13 years.^41^

These regional successes were important, and demonstrate the ongoing interest of many local Association leaders in positioning the rights of the gifted as an equalities issue. Yet, they were heavily dependent on support from local education authorities – and this was not widespread across Britain. As a consequence, it seems likely that much Association voluntary activity was predominately organized and attended by middle-class families. Qualitative suggestions of affluence are evident in the rich descriptions of clubs, often run from volunteers’ homes, as situated in, for example, ‘eight acres of rough pasture, a small, shabby farmhouse and [with] an assortment of domestic and farm animals’, or ‘a large estate on the borders of Surrey and Hampshire’.^42^ Gendered dynamics are also suggested in these rich accounts – while focusing on children’s activities and experiences, they also mention, for example, mothers as key organizers, ‘temporarily running’ such groups until children were ‘old enough for her to go back to primary school teaching’.^43^

The only quantitative evidence we have touching on Association demographics fortifies these qualitative impressions about class. A 1987 survey, sampling 125 families, concluded that the group’s membership remained disproportionately ‘middle-class’. While ‘middle-class’ was a broad category in this period, the survey also stated that its sample was ‘well-educated’ overall, and that only 6 per cent of their sample parents worked in manual professions.^44^ Indeed, the survey stated that the Association had ‘not yet succeeded in attracting as many members from the working classes’.^45^ The survey did not discuss race or ethnicity. Further, no available archival evidence suggests interactions between the National Association and the supplementary schools movement, organized by Black parents in this period to provide extra classes for their under-stretched children – despite the overlapping concerns and plans of these groups.^46^ While the Association did not use psychometric testing, such tests had contributed to a cultural stereotype of ‘gifted’ young people as associated with whiteness; in line with historic discourses around nation-building and the young as ‘good citizens’ in building empires.^47^ Such cultural stereotypes will have likely affected whether minoritized families felt comfortable accessing groups framed around ‘giftedness’– unless significant efforts were made by local organizers to frame these spaces inclusively.

Remaining Association files speak more about the motivations of parent-organizers than about their demographics. Letters and accounts from parents position their involvement emotionally, and as a necessary action to replace the poor provisions of the welfare state. Indeed, involved families spoke on television, in diaries, to Mass Observation, and to the Association about facing marital difficulties due to the strains of having an unhappy gifted child, and about being worried about their child being bullied and unhappy, all as they were not given adequate provision in mainstream schooling.^48^ Parents then framed their involvement with the Association around isolation and desperation: they were looking for help coping with a child they could not understand, and they were ‘near snapping point’.^49^ Amidst broader critique of the welfare state in the 1970s, parents framed this explicitly as a failure of education, social services, psychology, and associational life to cater for their distinct needs. One parent wrote to the National Association in 1977 that, ‘If I felt the need [for such provision], I reasoned, others did too, so if no-one else was prepared to fill the gap why not do it myself?’^50^ Parents emphasized that filling this ‘gap’ was difficult – they were also ‘bring[ing] up four young children’ and needed ‘determination and sense of purpose’ – but they also felt that such voluntary action was necessary, for their own emotional well-being and that of their children.^51^ Parents, then, conceptualized collective action as the appropriate response to individual suffering; bringing together historic traditions of voluntary action with new articulations of individual experience and emotion.

## III

Association weekend and holiday clubs were structured by adult organizers around a specific vision of what gifted young people would need and want. The activities offered assumed that gifted children would be particularly physically, as well as intellectually, active, and engaged with practical, as well as intellectual, pursuits. They would, as a consequence, require particularly specialist and distinctive provisions, blending technical, academic, and sporting pursuits in ways that went beyond existing provision in schools.

The activities offered to the gifted young were often focused on the outdoors. Ideas of physical activity, mobility, and curiosity were embedded in the title of the courses, positioning young people as ‘Explorers’, suggestive of their physical adventurousness, as well as their intellectual prowess. Holiday camps would include active games such as ‘Hide and Seek, Capture the Flag etc’.^52^ These outdoor activities were part of a vision of ‘good’ rural citizenship, with nature as a ‘facilitator of a morally and physically healthier citizen’, as described by Sian Edwards and Siân Roberts.^53^ The rural homes that provided space for the holiday camps, indeed, were said to offer participants ‘the true sense of values which comes from an appreciation of the outdoors’.^54^ Magazines also looked to inform young readers about wildlife rescue, how to identify garden birds, and ‘Understanding the countryside’.^55^ This emphasis on the outdoors reiterated a post-war vision of the countryside as ‘traditional…a symbol of a timeless and shared national past’; quite separate from the simultaneous 1960s and 1970s moral panics around urban youth cultures of juvenile delinquency and gangs.^56^

The clubs also offered a variety of inside activities. One description stated that clubs offered creative arts, intellectual debate, drama, computer science, mineralogy, and chess.^57^ Another, emphasizing how activities sprawled across an organizer’s home, discussed ‘woodwork in the basement, photography in the airing cupboard – in the hall, cooking, all kinds of games, maths, logic, chess and the more mundane kinds in the dining room’.^58^ The precise nature of activities offered in gifted youth culture are difficult to characterize and describe. Something notable, however, is that these activities offered sporting, academic, manual, and creative outlets. This was distinct from the separate cultures created by grammar and technical school curricula in the post-war period, where the former focused on academic materials and the latter manual ones, with minimal crossover.^59^ Gifted youth culture offered academic pursuits from the curriculum – such as maths – and beyond it – such as logic, debate, computer science, and drama. It also offered the more technical ‘life skills’ of cooking, creative arts, and the mysteriously described ‘mundane kinds’. This range of activities presupposed that gifted young people would benefit from being stretched in multiple ways, and that the existing provisions of the welfare state were inadequate.

The distinctive nature of gifted youth culture becomes yet more visible through analysis of the National Association’s youth magazines. These magazines were segmented for children aged 3–7, 7–12, and 12–17, and were typically around twenty to thirty pages long. They primarily contained children’s own drawings, poetry, book reviews, requests for pen pals, advertising for exhibitions and books, and views on topics such as education, though the adult editor also sometimes added articles expected to be of interest, and engaged in some dialogue with young correspondents. Most child readers published only one contribution, but those who wrote in often referenced older issues and discussions. This suggests that the magazines had a long-standing and significant audience, who would also meet at times at face-to-face events, but that they did not forge a continuous epistolary community within the space of the magazine itself. Notably, here, in the writings of the young people and articles suggested by the editor, scientific developments were frequently discussed, particularly archaeology, computers, and the sciences of measurement such as skulls and toe size.^60^ Space was also a common theme, and magazines showed youth clubs observing rocket launches, space agencies sending information to schools, and reviews of books about ‘Tomorrow’s world’.^61^

Many themes were absent from these youth magazines. Notably, for example, the magazines’ ‘political forum’ did not typically discuss party politics or contemporary social movements such as second-wave feminism. Rather, they focused on scientific and economic policy, discussing nuclear defence and the ‘brain drain’.^62^ Discussions of popular culture and society in these magazines did not consider contemporary issues of youth sexuality, consumption, fashion, or music. Rather, magazines focused on more niche pursuits: ephemera, ‘New Images of Man’ in post-war French art, Tolkien books, and fantasy gaming.^63^ Historical discussions focused primarily on ‘dinosaurs & pre-history’, evolution, stately homes, the English Civil War, and the American Revolution.^64^ This constellation of areas constructed ‘gifted youth culture’ in distinct ways, removed from permissive youth cultures of the same period.

Instead, adult-constructed spaces for gifted youth culture sought to draw the young gifted together, and thus away from their ‘non-gifted’ peers. A 1978 newsletter, reporting on a holiday retreat in Monmouth, Wales, stated that young people attending ‘enjoy a week spent in a world in which they are normal…they rarely get the chance to play with children of similar ability’.^65^

The ideas of youth sociability underpinning this were not distinct to gifted spaces alone. Laura Tisdall has shown how, from the 1950s, psychology and education demanded that the ‘normal child’ must exhibit ‘extroversion and sociability’, rather than living the ‘inter-war ideal of a quiet, well-behaved individual’.^66^ Nonetheless, the manifestation of this in 1970s and 1980s gifted youth culture was distinct. Notably, gifted youth were drawn together as a cohort of potential future leaders, whose leadership – and social value – would be based on intellect.

Ideas of preparing young people for their future roles were frequent in Association magazines. In its Summer 1979 bulletin, the Association asked children to think about the fact that: ‘THE YOUNG PEOPLE OF THIS COUNTRY HAVE A GREAT DEAL TO OFFER TO HELP THOSE LESS FORTUNATE THAN THEMSELVES – BOTH WITH IDEAS AND DIRECT HELP.’^67^ The magazine asked children to undertake projects on key themes. One theme was war, and the magazine stated: ‘One day a “small” war could grow into the nightmare of total devastation by nuclear weapons. What can we do to begin to lessen the risks and start building a lasting peace?’^68^ Another key theme was hunger: ‘How can we achieve a sense of unity in the world when some of us have so much and others lack the basic necessities?’^69^ Another, freedom: ‘All over the world the basic human rights are being denied because of race, colour or belief. In Britain, freedom is being eroded. What can we do to turn the tide?’^70^ While sadly no traces remain of children’s responses to these prompts, their existence nonetheless demonstrates the adult interest in shaping the gifted young as distinct future leaders.

Gifted youth culture would equip Association members to meet these vast challenges in a variety of ways. The Association’s Bristol and Isle of Wight Saturday Clubs, for example, taught gifted children Esperanto, a language created for international use. One teacher in Bristol told the Association’s April 1978 newsletter that, ‘in taking up Esperanto one takes up a key to world understanding and to world harmony’.^71^ Other Association magazines advertised ‘Project Trident’: a ‘year between scheme’ enabling young people who left school at eighteen to participate in voluntary service, work away from home, and take ‘challenging Outward Bound courses, Brathay expeditions or Ocean Youth Club sailing cruises’.^72^ These experiences would show gifted students, before they entered university or established careers, ‘how and where they fit in the wider community and what it means to be trusted, respected, needed by and important to other people’.^73^ The Association also helped children to identify and communicate with pen pals from America and Australia, looking to develop conversations ‘which might eventually spread all over our beautiful little world’.^74^ All of these diverse schemes reminded the gifted young that they were distinctive individuals, and that they had a role to play, today and in the future, in forging a better world. This continued and extended the efforts of interwar voluntary organizations, such as the League of Nations Union. The Association continued to push a vision of ‘world citizenship’, but responded also to the ‘new challenges’ of the late twentieth century. The Association also focused on influencing a distinct group of future leaders, rather than shaping all children as future citizens, reflecting and embedding popular individualism at this time.^75^

Thus, Association spaces relied on the assumption that gifted young people required a distinctive cultural space: offering physical and intellectual pursuits; interests in science, rural life, and high culture; and shared socializing with new peers. This offering was detached from the political and cultural shifts of the 1960s, 1970s, and 1980s. It was not a space of punk music, milk bars, new cinema, or satirical comedy. Nor was this space characterized by, for example, simultaneous developments in youth culture such as the opening of new nightclubs.^76^ Nonetheless, this space was also not necessarily a ‘vanguard’ against the ‘perceived threat of “modernity”‘; organizers made no explicit reference, in their writings, to concern about permissiveness or concurrent youth cultures.^77^ The spaces constructed for gifted youth are better understood within the blending of older traditions of collective action and newer ones of popular individualism in the 1970s and 1980s in Britain: parents felt that their young people required particularly distinct forms of leisure and social life, as individuals, but also that the solution to these new needs was small-scale collective action.

## IV

Young people engaged in complex ways with Association spaces. Many accepted the idea that they required distinctive forms of youth culture, and embraced this, bringing it to bear on interactions with school peers. As theoretical literature on agency by Susan Miller and Tatek Abebe emphasizes, agency can mean assent, as well as critique.^78^ Yet, young people were not passive recipients of these spaces – they also modified them through conscious criticism of adult ideas and through their everyday interactions.

This comes into clearer view through a case-study of one particularly long-standing National Association club: the Moberly Saturday Club, in north-west London. The roots of this club developed in 1971, when two parents established the London Explorers’ Club in their home. As was typical, the club enabled attending young people to pursue academic knowledge acquisition games and puzzles alongside a range of crafts, arts, and manual activities. From 1976, after a partnership with the Inner London Educational Authority was established, the group’s activities moved from a house to the Moberly Centre. This fostered significant expansion and, by November 1978, the National Association newsletter reported that the group served up to 150 Explorers each Saturday afternoon in term-time.^79^ This shift also saw a formalization and professionalization of the Club. The newsletter stated that the group had employed a part-time professional director, relatively atypical for such groups, and that its initial founder was also available to give advice for similar projects.^80^

As Kathryn Gleadle and Ryan Hanley have demonstrated, we can read children’s agency even from adult-produced sources, with careful attention.^81^ Accounts of adult organizers of the Moberly Club show that young people engaged in a variety of ways, constructing imaginative play that went beyond planned activities. Discussing the home-based version of the club, in a 1976 newsletter, one parent-organizer wrote that ‘in the bathroom’ of the house ‘extraordinary sex changes occurred – boys appearing mincing down the stairs having found the Chairman’s teenage daughter’s nightgown, wig and lipstick, and girls appeared with moustaches in navy uniform’.^82^ While tempting to read this as an example of permissiveness and fluid gender norms, scholarship tells us also that such playing with gender, using cross dressing as entertainment, has often reinforced gender norms as much as challenged them.^83^ Nonetheless, it is significant that involved young people used household objects from spaces not designated for formal play, and that they led their own activities. Other parent accounts of the Moberly group recall an atmosphere where: ‘No gasps of disapproval were heard by those wishing to spend the afternoon sliding down the banisters’ and children moved ‘at will from area to area’.^84^ Adults organizing these courses did not prevent, and indeed happily reported on, this later. Archival materials provide some evidence that this type of imaginative play may have been dampened by the move to the Moberly Centre in 1976. This new space was more managed and formalized, and the young people involved had to book and choose activities in advance, rather than using whatever household items they came across. One Association report stated that a ‘few’ of the ‘original’ members did not like ‘the new regime’, and that it had lost ‘intimacy’ and an ‘easy and free choice of things to do’.^85^ Nonetheless, other young Explorers testified to very much enjoying the new space, and the activities made available to them.^86^

Young people’s writings about the Moberly Club show that while engaging with this space in playful ways, they also accepted the broad ideas that they needed distinct provision as gifted young people, that they were different from other young people of their age, and that they were, potentially, future leaders. Two relevant testimonies were offered in a 1979 edition of the journal of the National Association, from a ten-year-old and a thirteen-year-old boy. Discussing the club, the ten-year-old stated that typically he joined in with school games played by his friends, but that ‘what I would really like to do also would be to talk about the world with them but no one wants to as they would all much rather play “catch”’.^87^ For this child, the Saturday Club meant that he had been able to meet an ‘older boy’, who ‘discusses with me everything I would like to know about the wealth of Great Britain, the world monetary system, and political parties and what they stand for’.^88^

The thirteen-year-old stated that when starting education at the age of five, he had already completed the relevant reading and ‘found school very uninteresting’. Unlike the ten-year-old boy, who was able to socialize with his classmates, the thirteen-year-old found that it was ‘difficult’ to mix socially as ‘there was little I could share with them’.^89^ As a consequence, the boy ‘found solace only in reading’ and was frequently ‘bored and restless’.^90^ When he met a psychologist, who ‘explained to me that I was “gifted”’, his life changed.^91^ For the following two years, he explained, he felt ‘timid and withdrawn’, though academically he continued to perform well. Aged nine he joined the Saturday Club and was able to socialize with new peers and to develop special interests in computers, photography, and philosophy. The boy stated that: ‘These things gave me something to work at and be proud of’, and also a peer group he could identify and socialize with.^92^ Overall, he felt now that he should try to offer something of ‘importance to the world around me’, particularly based on his ‘mind…this I feel I can offer to the world’.^93^ These young people then both testified that the label gifted had been very significant in their lives. It had changed how they felt about themselves and their peer groups, and also given them ideas of their own future roles in society. These young people’s sense of being a ‘gifted individual’ had been augmented through participation in Moberly activities.

Beyond the Moberly Club, these ideas were also common in the writings of young people to Association children’s magazines, and in their recorded testimonies from other local branches. The ideas of being a distinct group, ‘the gifted’, separate from other peer groups, come through strongly in transcribed recordings of two children attending the Wolverhampton branch – described by Association publications as situated in ‘an educationally deprived area’.^94^ In free-form conversation with an interviewer, ‘J’, a boy aged ‘11 plus’ and ‘B’, a girl aged ‘12 plus’ both emphasized that the identity of giftedness made them feel different from their peers at school.^95^ Significantly, these children embraced this. J stated that he, ‘enjoyed…being so far ahead of the other children’ and feeling ‘solitary’, while B said, ‘I’m becoming a “lone wolf” and I like it.’^96^ B even performed her distinct gifted identity in interactions with school peers, indeed, stating that, ‘I liked to invite school friends to tea then I got so bored with them that I asked for them to be taken home again.’^97^ The writings of gifted young people likewise frequently suggested that they saw themselves as distinct from teachers also, and found many teachers to be overly strict with them – a finding that chimes with Laura Tisdall’s argument, based on analysing teachers’ magazines, that gifted children in post-war England and Wales were ‘stereotyped as “cocky”’.^98^ Gifted young people then took the distinct ideas of gifted spaces in to their school environments.

Many young people also embraced the idea of themselves as future leaders, because of their gifted identities. This sense is clear in explicit policy commentary of young Association members – for example a sixteen-year-old writing to the Association in 1980 that Britain’s ‘biggest problem…at the moment is that the population is too large’: ‘if we were to divide the world’, they advised, ‘it should be done so that small groups emerge and so that everyone could participate in directing their state’.^99^ Another sixteen-year-old, likewise engaging with global politics, wrote to the Association in 1982 to criticize the nuclear deterrence defence system as ‘farcical’, given first that ‘many non-aligned countries have developed or could develop weapons of massive death potential’.^100^ In addition to seeing themselves as future leaders, the gifted young also expressed anxieties about the Cold War more generally, and in creative ways. In a 1979 edition of *Explorers unlimited*, a young person whose age was recorded as ‘13 or 14’ wrote a short horror story, in which a man in bed, hearing a plane fly overhead, thought: ‘Perhaps it was Russian. It might be carrying a bomb, an atomic bomb. There might be another war.’^101^ Such broad concerns about the Cold War have been traced in the accounts of other young people in this period also, and, indeed, here hint towards the broader social engagements of the gifted young, who will have discussed warfare and global politics at school and in their family homes. Nonetheless, the ideas of future leadership were a distinctive part of gifted youth culture, linked to an idea of the gifted as special individuals.

Young Association members then felt different from their peers. Yet, their sense of difference was not entirely shaped by adult conceptions. Rather, young members also changed adult-produced ideas about gifted youth culture. First, young people questioned the specific label ‘gifted’, and how it changed adult behaviours. The 1979 letter from American young people – with which this article opened – also stated that ‘being gifted does not make everything easy’; it made teachers ‘push harder’ and ‘others who are not gifted resent me sometimes for getting better grades or being just a little more perceptive than them’.^102^ British respondents wrote that they felt ‘different’, likewise, but argued that while teachers and peers recognized them as ‘gifted’, they did not receive special provision in school, but rather worked in environments without ‘much proper streaming’ where they missed out on ‘stimulating conversation, [and] a chance to exchange views and opinions’ with gifted peers.^103^ Young people then happily joined the National Association, and took advantage of the activities offered, but also questioned how broader educational and social structures framed ‘giftedness’. Gifted young people were also not afraid to criticize adult ideas more broadly – also in poetry, for example, criticizing adults for forcing them to travel on the London underground, despite their fears, or cruelly removing them from an enjoyable Christmas fair.^104^

A second theme which gifted young people reconceptualized was the idea of national leadership. The ten-year-old mentioned above, for example, had a complex relationship with visions of his future role. He stated that he would ‘like to become a barrister and a Member of Parliament as I find law and politics fascinating and I love talking’.^105^ In this account, his preferred imagined future – in that moment – was pursuing law or politics, potentially productive pathways for the gifted child. Nonetheless, the young person did not connect these professions with ideas of national or global morality, virtue, or social value. Rather, he would pursue these routes because he found them ‘fascinating’ and ‘love[d] talking’.^106^ Further surveys, discussed in Association newsletters, found that the gifted young wanted a diverse range of careers – not only in leadership positions but also as ‘a fashion writer’, ‘a professional dancer’, or managing ‘a travel agency’.^107^ The gifted young were confident in their future prospects, but wanted to determine how they would use their ‘gifts’; they did not accept the characterization of themselves as ‘future leaders’ lightly.

A third idea that the gifted young modified was the assumption that they would want to socialize together. In this case, young members modified this idea through their lived behaviours, rather than through explicit critique. Indeed, Association members often experienced peer disputes and conflict on Association courses, shaped by a powerful sense of age and gender distinction. A description from a parent-organizer of a camp in South Wales in 1977, for example, emphasized that gifted youth brought into contact with one another struggled if competitions were not won and lost aligned with chronological age. The author described how one ‘sturdy nine-year-old’, ‘took it very hard that he could be beaten at target shooting by a weedy seven-year-old’.^108^ More broadly, this organizer also described how attendees had often not played with other gifted youth before and, hence, ‘were not used to competition. They were used to winning. And they did not much like the new experience of being beaten.’^109^ This focus on chronological age as a marker of difference was furthered in Association magazines, where children wrote to the group with brief notes that they were, for example, not merely aged nine but ‘9¾’.^110^ The gifted young, again, enthusiastically participated in leisure spaces for ‘the gifted’, but pre-existing cultural ideas about how capacity *should* relate to age and gender were not entirely over-ridden by this new identity. Children’s interest in chronological age likely came from their concurrent experiences in schools, where this concept had shaped the ‘practical reorganisation of schooling’ since the 1930s, and increasingly since the Second World War.^111^ A sense of age must be included in our understandings of popular individualism.

The gifted young then exercised agency: many assented to the idea that they had unique capacities as distinctive individuals, but they also made claims about how this should affect their future roles, and adult and peer relationships.^112^ The gifted young reshaped gifted youth culture, and their accounts must be critical to understanding it. They brought assumptions from spaces designed for ‘the gifted’ into schools (notably ideas of their own intellectual superiority) and they brought assumptions from their broader lives into gifted spaces (particularly around the significance of age). Gifted youth spaces were thus a part of the rich tapestry of these young people’s social and cultural lives, but spaces that presented a powerful vision of distinctiveness and difference.

## V

The mid- to late twentieth century in Britain saw an explosion of new medical and social categorizations and classifications, buoyed by new forms of writing and the development and formalization of new professions. This period also saw a series of cultural, social, and political changes around permissiveness and the ‘new society’, which reshaped the lives of the young economically, socially, and politically. This article uses a seemingly small case-study – the leisure activities of those identified as ‘gifted’, as co-ordinated by the National Association for Gifted Children – to illuminate what these trends meant and how they were experienced.

Giftedness was a label of great power and privilege: it was tied up historically with eugenicist ideas and significant discrimination. Yet, parents involved in giftedness organizations emphasized that they joined not to gain power nor prestige, but rather due to their experiences and emotions of desperation and isolation. Notably, these parents, mobilizing in the 1970s and 1980s, felt that their families had fallen through ‘gaps’ in the post-war welfare state, illustrating Robinson, Schofield, Sutcliffe-Braithwaite, and Thomlinson’s argument that the welfare state was itself one of the ‘diverse drivers’ for the rise of popular individualism in the 1970s: for it ‘promised equality (even if in paradoxical ways)’ and thus shaped subsequent popular accounts of individual rights.^113^

Critically, these parents believed that a universal welfare state should provide for the distinct individual needs of all young people, and also that existing systems of education and welfare were failing to do so. Their response was not to lobby for systematic change, nor to do nothing, but to meet these requirements themselves, through voluntary action, establishing new leisure spaces specifically for ‘the gifted young’ across Britain. Many of these clubs tried to include working-class and ethnic minority populations, but overall they were likely dominated by white, middle-class families. Inside the clubs, adult-organized activities told the involved young that they were special and unusual, and that they must interact with one another.

The gifted youth cultures created by the National Association branches look far removed from the permissive cultures often charted by existing historical and sociological accounts.^114^ Young people did not engage with new fashions, music, or consumer trends in these spaces, but rather with classical music and history, outdoor pursuits, and practical skills such as woodwork. Association magazines did not condemn or criticize permissive cultures; they simply did not mention them. This then contributes to our thinking about the significance of permissiveness in daily life: if the permissive youth cultures of the 1960s were this invisible in one youth space by the 1970s, where else were they absent, and how powerful can they have been? Yet, this characterization also goes beyond Mills’s account of many youth lives as ‘mundane’ or ‘ordinary’, rather than permissive, in these decades.^115^ The gifted young were seen as far from ordinary – they were presented as potential future leaders, albeit in fuzzily characterized ways. Youth cultures were not merely ‘mundane’ or ‘permissive’ in the mid- to late twentieth century; they were individual. Youth cultures were segmented in an endless variety of distinct ways, as localized and community action looked to cater for all preferences and needs. Historiographies of youth culture must account for individualism, and descriptions of popular individualism must account for age.

Young people themselves shaped youth cultures too. This statement should not seem controversial: since the 1990s, historians of childhood have written powerful accounts of childhood action.^116^ Yet, nonetheless, this field remains seen as ‘on the margins’ of historical scholarship, according to recent critical historiographical accounts, which attribute this to young people’s limited ‘power’ and to a lack of available sources.^117^ This article has looked to rebuff these claims. It has shown that the gifted young had ‘power’. Using recent methodological thinking about ‘agency’, which shows how agency can be assent, or small-scale change, it has demonstrated that the young changed the spaces of gifted youth culture both through conscious critique and by virtue of how they engaged with available activities.^118^ The article has also shown that we do have sources available through which to understand children’s lives in the past, by both reading ‘against the grain’ of adult-accounts – as Gleadle and Hanley have also demonstrated – and also through young people’s own writings, which were accessed by working with a voluntary organization to access rich, but uncatalogued materials.^119^ This work often simply requires going beyond existing established archives.

Overall, then, the case-study of this article seems small – giftedness was not a significant part of the welfare state, or of national educational provision, in modern Britain, and thus has been excluded from existing historical accounts. Yet, there were rich and distinct spaces of youth culture created around this category by passionate campaigners and parents. Tracing the assumptions embedded in these spaces, and youth responses to them, shows the diversity of ‘youth cultures’ in modern Britain. Even this niche subgroup, ‘the gifted young’, received distinct provision in these decades, and expected to be treated as distinct individuals due to this new identity. Even in these spaces, which were small scale and seem very specific, there were also significant tensions around what ‘gifted youth culture’ meant. This then shows the pervasiveness and power of ideas about ‘individualism’ in daily life in 1970s and 1980s Britain. It shows also that the tensions around theoretical and political constructions of ‘the individual’– examined in existing historiographical accounts – were also reshaped in daily life.^120^ Critically, this article has furthermore demonstrated that gifted youth culture was significantly influenced by the young themselves, and thus that the agency of young people must be a critical part of our historical accounts. New groups of young people felt increasingly ‘individual’ in the 1970s and 1980s; their resulting actions drove voluntary action and reshaped cultures of leisure, education, and sociability in daily life.

## Footnotes

1 National Association for Gifted Children archives, Bletchley Park (hereafter NAGC), *Explorers unlimited* newsletter, Summer 1979, ‘What’s it like then?’, p. 1.

2 Ibid., Autumn 1979, ‘Education forum: what’s it like then?’, p. 1.

3 Sally Shuttleworth, *The mind of the child: child development in literature, science, and medicine, 1840–1900* (Oxford, 2010); Lewis Terman, *Mental and physical traits of a thousand gifted children* (Stanford, CA, 1925); Jon Agar, *Science in the twentieth century and beyond* (London, 2013), pp. 73, 105; Alexandra Minna Stern, *Eugenic nation: faults and frontiers of better breeding in modern America* (Oakland, CA, 2015).

4 Kirsten Gregory, ‘Exceptional and destructive: the dangerous child and the atom bomb in post-war science fiction’, in Monica Flegel and Christopher Parkes, eds., *Cruel children in popular texts and cultures* (London, 2018), pp. 153–72; Hans Staats, ‘“Tag…you’re it”: Cold War comics and the performance of boyhood and criminality’, in Flegel and Parkes, eds., *Cruel children*, pp. 173–91; Laura Tisdall, ‘“We have come to be destroyed”: the “extraordinary” child in science fiction cinema in early Cold War Britain’, *History of the Human Sciences*, advance access 2 Feb. 2021.

5 Jennifer L. Jolly, ‘The National Defense Education Act, current STEMP initiative, and the gifted’, *Gifted Child Today*, 32 (2009), pp. 50–3; Charles J. Russo, ‘Unequal educational opportunities for gifted students: robbing Peter to pay Paul?’, *Fordham Urban Law Journal*, 29 (2001), p. 734; Bodleian Library (hereafter Bod.), Joy Gibson and Prue Chennells, eds., *Gifted children: looking to their future* (Essex: The Anchor Press, 1976), pp. 20–34.

6 ‘Briefing paper: grammar schools’, Secondary Education and Social Change Project, Jan. 2018, https://sesc.hist.cam.ac.uk/wp-content/uploads/2018/01/Briefing-paper-Grammar-Schools.pdf (13 Sept. 2021), p. 1. See also Jacky Brine, ‘Tales of the 50-somethings: selective schooling, gender and social class’, *Gender and Education*, 18 (2006), pp. 431–46; Richard Harris and Samuel Rose, ‘Who benefits from grammar schools? A case study of Buckinghamshire, England’, *Oxford Review of Education*, 39 (2013), pp. 151–71; Diane Reay, ‘The Zombie stalking English schools: social class and educational inequality’, *British Journal of Educational Studies*, 54 (2006), pp. 288–307.

7 John Parker, ‘Education Bill’, House of Commons, 19 Jan. 1944, vol. 396, col. 233; Hansard, House of Lords, Education (Scotland) Bill, 6 June 1945, vol. 136, col. 435. Tabloid critique of this emerged in the 1970s and 1980s, see Christopher Rowlands, ‘Why gifted Claire is a misfit’, *Daily Mail*, 9 Aug. 1978, p. 6; Graham Turner, ‘Full of eastern promise’, *Daily Mail*, 23 June 1989, p. 32; Ray Massey and Ray Honeyford, ‘A dream of genius’, *Daily Mail*, 16 Apr. 1991, p. 32; Ian Smith, ‘Treated like a freak for being clever’, *Daily Mail*, 28 Nov. 1978, pp. 6–7.

8 S. A. Bridges, ed., *Gifted children and the Brentwood experiment* (London, 1969); Bod., Oxfordshire Education Committee, *Exceptionally gifted children* (Oxford: Oxfordshire Education Committee, 1956?), p. 1. Quote is from Department of Education and Science, *Her Majesty*’*s Inspectorate Series, matters of discussion: gifted children in middle and comprehensive secondary schools: a discussion paper by a working party of Her Majesty*’*s Inspectorate* (London: HMSO, 1977), www.educationengland.org.uk/documents/hmi-discussion/gifted.html (16 Dec. 2020), p. 13.

9 *Excellence in schools*, July 1997, Cm 3681, www.educationengland.org.uk/documents/wp1997/excellence-in-schools.html (17 Nov. 2020); *Schools achieving success* (Cm 5230, 2001), https://assets.publishing.service.gov.uk/government/uploads/system/uploads/attachment_data/file/355105/Schools_Achieving_Success.pdf (28 July 2021). Forthcoming analysis of this in Jennifer Crane, *Equal elites: gifted children in Britain and the world, 1945*–*2010* (in preparation).

10 Absent, for example, in Peter Mandler, *The crisis of the meritocracy: Britain*’*s transition to mass education since the Second World War* (Oxford, 2020); Pat Thane, *Divided kingdom: a history of Britain, 1900 to the present* (Cambridge, 2018); Mathew Thomson, *The landscape of the child and the British postwar settlement* (Oxford, 2013).

11 Mary Stott, ‘In April’, *Guardian*, 6 Apr. 1967, p. 6.

12 Oliver Pritchett, ‘The loneliness of the too-gifted child’, *Guardian*, 20 Mar. 1966; ‘Serious lack of facilities for creative children’, *Guardian*, 21 Mar. 1966, p. 2; ‘New body aims to get help for the gifted child’, *Times*, 18 June 1966.

13 NAGC, annual report 1981, p. 7.

14 Mark Abrams, *The teenage consumer* (London, 1959); Michael Schofield, *The sexual behaviour of young people* (London, 1965); idem, *The sexual behaviour of young adults: a follow up study to the sexual behaviour of young people* (London, 1973). See also Axel Schildt and Detlef Siegfried, ‘Youth, consumption, and politics in the age of radical change’, in Axel Schildt and Detlef Siegfried, eds., *Between Marx and Coca-Cola: youth cultures in changing European societies 1960*–*1980* (London, 2006); Jeffrey Weeks, *The world we have won: the remaking of erotic and intimate life* (Abingdon, 2007); Arthur Marwick, *The sixties: cultural revolution in Britain, France, Italy and the United States, c. 1958* – *c. 1974* (Oxford, 1998); Bill Osgerby, *Youth in Britain since 1945* (Oxford, 1998); John Springhall, *Youth, popular culture and moral panics: Penny Gaffs to Gangsta-Rap 1830*–*1996* (Basingstoke, 1998).

15 On radical lives, see Celia Hughes, ‘Young socialist men in 1960s Britain: subjectivity and sociability’, *History Workshop Journal*, 73 (2012), pp. 170–92; idem, *Young lives on the left: sixties activism and the liberation of the self* (Manchester, 2015). On moral panic, see Stanley Cohen, *Folk devils and moral panics: the creation of the Mods and Rockers* (London, 1972); Bill Osgerby, ‘The good, the bad and the ugly: postwar media representations of youth’, in Adam Briggs and Paul Cobley, eds., *The media: an introduction* (Harlow, 1997); Angela Bartie, ‘Moral panics and Glasgow gangs: exploring “the new wave of Glasgow hooliganism”, 1965–1970’, *Contemporary British History*, 24 (2010), pp. 385–406; Louise Jackson and Angela Bartie, *Policing youth: Britain, 1945*–*1970* (Manchester, 2014). For accounts that acknowledge mundanity, see Schofield, *The sexual behaviour of young people*; idem, *The sexual behaviour of young adults*; Pearl Jephcott, *Time of one*’*s own: leisure and young people* (Edinburgh, 1967); Jim Gledhill, ‘White heat, guide blue: the Girl Guide movement in the 1960s’, *Contemporary British History*, 27 (2013), pp. 65–84; Helena Mills, ‘The experience and memory of youth in England c. 1960 – c. 1969’ (D.Phil. thesis, Oxford, 2016), https://ora.ox.ac.uk/objects/uuid:f0bdc321-b580-414d-a7ff-35d2c92e3ad4 (4 June 2021), pp. 2, 6.

16 Sian Edwards, ‘“Nothing gets her goat!” The farmer’s wife and the duality of rural femininity in the Young Farmers’ Club movement in 1950s Britain’, *Women*’*s History Review*, 26 (2017), p. 27.

17 Aled Davies, James Freeman, and Hugh Pemberton, ‘“Everyman a capitalist” or “free to choose”? Exploring the tensions within Thatcherite individualism’, *Historical Journal*, 61 (2018), pp. 477–501; Florence Sutcliffe-Braithwaite, *Class, politics, and the decline of deference in England, 1968*–*2000* (Oxford, 2018), pp. 4–8.

18 Jon Lawrence, *Me, me, me? The search for community in post-war England* (Oxford, 2019), p. 2; Emily Robinson, Camilla Schofield, Florence Sutcliffe-Braithwaite, and Natalie Thomlinson, ‘Telling stories about post-war Britain: popular individualism and the “crisis” of the 1970s’, *Twentieth Century British History*, 28 (2017), pp. 268–304.

19 Mona Gleason, ‘Avoiding the agency trap: caveats for historians of children, youth, and education’, *History of Education*, 45 (2016), pp. 446–59; Lynn M. Thomas, ‘Historicising agency’, *Gender & History*, 28 (2016), pp. 324–39; Susan A. Miller, ‘Assent as agency in the early years of the Children of the American Revolution’, *Journal of the History of Childhood and Youth*, 9 (2016), pp. 48–65; Tatek Abebe, ‘Reconceptualising children’s agency as continuum and interdependence’, *Social Sciences*, 8 (2019), pp. 1–16.

20 Chris Millard, ‘Using personal experience in the academic medical humanities: a genealogy’, *Social Theory & Health*, 18 (2019), pp. 184–98.

21 Heather Shore, ‘Youth culture in modern Britain, c. 1920 – c. 1970: from ivory tower to global movement – a new history’, *Cultural and Social History*, 7 (2010), pp. 280–4. A notable exception is Jane Deverson and Charles Hamblett, *Generation X* (London, 1964).

22 Colin Heywood, ‘On the margins or in the mainstream? The history of childhood in France’, *Nottingham French Studies*, 59 (2020), p. 135; Hendrick in Carmel Smith and Sheila Greene, eds., *Key thinkers in childhood studies* (Bristol, 2015), p. 121; Sarah Maza, ‘The kids aren’t all right: historians and the problem of childhood’, *American Historical Review*, 125 (2020), pp. 1261–85. See also the critical responses to Maza’s article in this special issue.

23 Looking at the nineteenth and early twentieth centuries, see Sian Pooley and Colin Pooley, ‘“Such a splendid tale”: the late nineteenth-century world of a young female reader’, *Cultural and Social History*, 2 (2005), pp. 329–51; Siân Pooley, ‘Children’s writing and the popular press in England, 1876–1914’, *History Workshop Journal*, 80 (2015), pp. 75–98; Laura Tisdall, ‘“That was what life in Bridgeburn had made her”: reading the autobiographies of children in institutional care in England, 1918–1946’, *Twentieth Century British History*, 24 (2013), pp. 351–75; Catherine Sloan, ‘“Periodicals of an objectionable character”: peers and periodicals at Croydon Friends’ School, 1826–1875’, *Victorian Periodicals Review*, 50 (2017), pp. 769–86; Rebecca Swartz, ‘Children’s experiences of the Children’s Friend Society emigration scheme to the colonial Cape, 1833–41: snapshots from compliance to rebellion’, in Sian Pooley and Jonathan Taylor, eds., *Children*’*s experiences of welfare in modern Britain* (London, 2021), pp. 27–48; Gillian Lamb, ‘“Their mother is a violent drunken woman who has been several times in prison”: “saving” children from their families, 1950–1900’, in Pooley and Taylor, eds., *Children*’*s experiences of welfare*, pp. 49–72; Claudia Soares, ‘“Dear Sir, remember me often if possible”: family, belonging and identity for children in care in Britain, c. 1870–1920’, in Pooley and Taylor, eds., *Children*’*s experiences of welfare*, pp. 73–98; Sian Pooley, ‘Child philanthropy, family care and young bodies in Britain, 1876–1914’, in Pooley and Taylor, eds., *Children*’*s experiences of welfare*, pp. 99–122. Looking at the mid- to late twentieth century in this area, see Sarah Kenny, ‘“Basically you were either a mainstream sort of person or you went to the Leadmill and The Limit”: understanding post-war British youth culture through oral history’, in Kristine Moruzi, Nell Musgrove, and Carla Pascoe Leahy, eds., *Children*’*s voices from the past: new historical and interdisciplinary perspectives* (London, 2019), pp. 233–59; Jonathan Taylor, ‘“The borough council have done a great deal…I hope they continue to do so in the future”: children, community and the welfare state, 1941–1955’, in Pooley and Taylor, eds., *Children*’*s experiences of welfare*, pp. 147–74; Tisdall, ‘“The school that I’d like”: children and teenagers write about education in England and Wales, 1945–1979’, in Pooley and Taylor, eds., *Children*’*s experiences of welfare*, pp. 197–220; Caroline Rusterholz, ‘Teenagers, sex and the Brook Advisory Centres, 1964–1985’, in Pooley and Taylor, eds., *Children*’*s experiences of welfare*, pp. 247–72.

24 The Education Committee of the National Association for Gifted Children, *Help with bright children* (London: National Association for Gifted Children, 1989), p. 16.

25 E. Susanne Richert, ‘Identification of gifted children in the United States: the need for pluralistic assessment’, *Roeper Review*, 8 (1985), pp. 68–72; E. Susanne Richert, ‘Rampant problems and promising practices in the identification of disadvantaged gifted students’, *Gifted Child Quarterly*, 31 (1987), pp. 149–54; Donna Y. Ford, ‘The underrepresentation of minority students in gifted education: problems and promises in recruitment and retention’, *Journal of Special Education*, 32 (1998), pp. 4–14; J. John Harris and Donna Y. Ford, ‘Hope deferred again: minority students underrepresented in gifted programs’, *Education and Urban Society*, 31 (1999), pp. 225–37.

26 Rob Waters, *Thinking Black: Britain, 1964*–*1985* (Oakland, CA, 2018). See also Steve McQueen’s *Small axe* episode on Education (2020) and the BBC’s documentary *Subnormal* (2021).

27 NAGC, *Looking to their future: the newsletter of the National Association for Gifted Children*, June 1976, ‘Margaret Branch’, p. 1.

28 NAGC, *Looking to their future*, Nov. 1978, ‘A study of attitudes, expectations and abilities within an NAGC branch’, p. 1.

29 NAGC, *Looking to their future*, June 1976, ‘Margaret Branch’.

30 Email correspondence with editor of these magazines, 28 Oct. 2021.

31 NAGC, *Looking to their future*, Nov. 1980, ‘National Association for Gifted Children: location of branches’, p. 12.

32 NAGC, newsletter, Autumn 1971, ‘Concentrating on the centres’, pp. 1–8.

33 Ibid., pp. 1–8.

34 Ibid., p. 1.

35 NAGC, *Looking to their future*, Nov. 1978, ‘A study of attitudes, expectations and abilities within an NAGC branch’, p. 1.

36 ‘“Miracle” view of gifted children criticized’, *Times*, 9 Nov. 1970, p. 2; Bruce Kemble, ‘Talent spotting begins at home’, *Daily Express*, 24 Mar. 1971, p. 6.

37 ‘Crime risk warning on gifted kids’, *Evening Post*, 6 Sept. 1975, p. 3; Sue Fisher, ‘Children with a special gift…’, *Mercury*, 25 Apr. 1975, p. 11.

38 NAGC, newsletter, May 1969, ‘Wolverhampton’, p. 5; NAGC, ‘Chairman’s report’, *Twelfth annual general meeting held at the Royal Institution of Saturday, 3 November 1979*, p. 1; NAGC, *Looking to their future*, Mar. 1979, ‘Gifted children and inner city areas’, p. 1; NAGC, newsletter, undated but inner material says 1984, ‘Recipe for success: Merseyside & Wirral’, p. 11; NAGC, branch newsletter, Mar. 1981, ‘Enrolment of less privileged children in NAGC activities’, p. 8. One involved organizer praised this scheme in a letter to *The Guardian*: Anne Crawford, ‘Waiting for action on mergers’, *Guardian*, 17 June 1980, p. 13.

39 NAGC, branch newsletter, Mar. 1981, ‘Enrolment of less privileged children in NAGC activities’, p. 8.

40 Ibid., p. 8.

41 NAGC, *Looking to their future*, Nov. 1980, ‘Liverpool’s Paddington III’, pp. 27–8; NAGC, branch newsletter, Mar. 1981, ‘Enrolment of less privileged children in NAGC activities’, p. 8; NAGC, ‘Chairman’s report’, *Twelfth annual general meeting held at the Royal Institution of Saturday, 3 November 1979*, p. 1.

42 NAGC, *Looking to their future*, May 1977, ‘Country holidays for young explorers’, p. 5; NAGC, *Explorers unlimited*, Mar. 1981, ‘Young adventurers’ camp, Summer 1981’, p. 5.

43 NAGC, *Looking to their future*, June 1976, ‘Practical support from the Inner London Education Authority’, p. 1.

44 Patricia Mason and Juliet Essen, *The social, educational, and emotional needs of gifted children* (London, 1987), p. 6. For discussion of the framework ‘middle-class’ in this period, see Sutcliffe-Braithwaite, *Class, politics, and the decline of deference*, ch. 2.

45 On definitions of class in this period, see Pat Thane, *Unequal Britain: equalities in Britain since 1954* (London, 2010), p. 4. Survey description: Mason and Essen, *The social, educational, and emotional needs of gifted children*, p. 3.

46 Waters, *Thinking Black*; Jacob Fairless Nicholson, ‘From London to Grenada and back again: youth exchange geographies and the Grenadian Revolution, 1979–1983’, *Antipode* (2020).

47 Alison Twells, *The civilising mission and the English middle class, 1792*–*1850: the* ‘*heathen*’ *at home and overseas* (Basingstoke, 2009); Kathryn Gleadle, ‘The juvenile enlightenment: British children and youth during the French Revolution’, *Past & Present*, 233 (2016), pp. 143–84; Stephen J. Heathorn, *For home, country, and race: constructing gender, class, and Englishness in the elementary school, 1880*–*1914* (Berlin, 2003); Jonathan Davies and Mark Freeman, ‘Education for citizenship: the Joseph Rowntree Charitable Trust and the educational settlement movement’, *History of Education*, 32 (2003), pp. 303–18; Peter Yeandle, *Citizenship, nation, empire: the politics of history teaching in England, 1870*–*1930* (Manchester, 2015).

48 ‘The bright, the backward…’, *Daily Express*, 3 Sept. 1974, p. 8; Stanley Bonnett, ‘Is there a genius in your family?’, *Daily Mirror*, 11 Sept. 1975, p. 7; The Keep, Mass Observation archives, G848, ‘Membership of the National Association for Gifted Children’, 18 Sept. 1984; British Film Institute, 492504, QED, *Challenging children: a gifted child*, 25 Sept. 1997; British Film Institute, *Open door: in with a head start?*, 7 Dec. 1978; British Film Institute, 79570, *Horizon: the gifted child*, 30 Jan. 1969.

49 NAGC, *Looking to their future*, June 1984, ‘Counselling’, p. 18. The significant impact of this counselling service for individual parents is discussed in *Looking to their future*, Nov. 1979, ‘A parent’s bird’s eye view on counselling’, p. 13.

50 *Looking to their future*, May 1977, ‘Country holidays for young explorers’, p. 5.

51 NAGC, newsletter, Autumn 1971, ‘Concentrating on the centres’, p. 1.

52 NAGC, *Explorers unlimited*, Mar. 1981, ‘Young adventurers’ camp, Summer 1981’, p. 5.

53 Edwards, ‘“Nothing gets her goat!”, pp. 26–45; Sian Edwards, *Youth movements, citizenship and the English countryside: creating good citizens, 1930*–*1960* (London, 2018); Siân Roberts, ‘Cultivating an “earthly paradise”: nature, informal education, and the contested politics of youth citizenship, 1910s–1940s’, *History of Education*, 49 (2020), pp. 498–516.

54 NAGC, *Explorers unlimited*, Mar. 1981, ‘Young adventurers’ camp, Summer 1981’, p. 5.

55 NAGC, *Dialogue*, Easter 1982, ‘Wildlife rescue service’, p. 16; NAGC, *Explorers unlimited*, Mar. 1981, ‘Young naturalists’, p. 32; NAGC, *Explorers unlimited*, Mar. 1981, ‘Explorer courses’, p. 5.

56 Edwards, ‘“Nothing gets her goat!”‘, p. 28.

57 Activities reported in June Tarlin, ‘Brain power just going to waste!’, *Daily Mail*, 16 Mar. 1976, p. 11; Barbara Jefery, ‘These children have a problem – they’re too intelligent!’ *Daily Mail*, 31 Jan. 1977, p. 10; Bod., Per 264505 e. 4, National Association for Gifted Children, *Journal of the Gifted Child*, Autumn 1979, inside leaf.

58 NAGC, *Looking to their future*, June 1976, ‘Practical support from the Inner London Education Authority’, p. 1.

59 See for more context ‘Briefing paper: grammar schools’, Secondary Education and Social Change Project; ‘Briefing paper: secondary modern schools’, Secondary Education and Social Change Project, Oct. 2017, https://sesc.hist.cam.ac.uk/wp-content/uploads/2018/02/Briefing-paper-Secondary-modern-schools.pdf (24 June 2017).

60 NAGC, *Dialogue*, Easter 1982, ‘Youth club rockets into space’, p. 11; NAGC, *Dialogue*, Easter 1982, ‘Discovering astronomy’, p. 12; NAGC, *Dialogue*, Easter 1982, ‘Skulls!’, p. 17; NAGC, *Questors Ho!*, issue 1–85, ‘How Toe-tal is the correlation?’, pp. 1–3; NAGC, *Explorers unlimited*, Mar. 1981, ‘Young astronomers’, p. 19; NAGC, *Explorers unlimited*, Mar. 1981, ‘Explorer courses’, p. 5; NAGC, *Explorers unlimited*, Mar. 1981, ‘Young archaeologists’; NAGC, *Explorers unlimited*, Mar. 1981, ‘Learning about nuclear energy’, p. 32; NAGC, *Explorers unlimited*, Summer 1979, ‘Computers, science’, p. 7.

61 NAGC, *Dialogue*, Easter 1982, ‘Youth club rockets into space, p. 11; NAGC, *Explorers unlimited*, Autumn 1979, ‘Tomorrow’s world’, p. 8.

62 NAGC, *Dialogue*, Easter 1982, ‘Political forum’, p. 9; NAGC, *Dialogue*, Easter 1982, ‘Education forum’, p. 19.

63 NAGC, *Dialogue*, Easter 1982, ‘Political forum’, pp. 22–3; NAGC, *Dialogue*, Easter 1982, ‘Aftermath: France 1945–54’, p. 9; NAGC, *Explorers unlimited*, Summer 1979, ‘Games workshop’, p. 7; NAGC, *Explorers unlimited*, Summer 1979, ‘The Tolkein Society’, p. 7.

64 NAGC, *Explorers unlimited*, Mar. 1981, ‘Explorer courses’, p. 5; NAGC, *Explorers unlimited*, Mar. 1981, ‘Dinosaur page’, p. 6; NAGC, *Explorers unlimited*, Summer 1979, ‘National Trust’, p. 5; NAGC, *Explorers unlimited*, Summer 1979, ‘Evolution and elephants’, p. 11; NAGC, *Explorers unlimited*, Autumn 1979, ‘The American Revolution’, p. 4; NAGC, *Explorers unlimited*, Summer 1979, ‘Puritan + Cavalier’, p. 4.

65 NAGC, *Looking to their future*, Apr. 1978, ‘Holiday retreat in Wales’, p. 9.

66 Laura Tisdall, *A progressive education? How childhood changed in mid-twentieth-century English and Welsh schools* (Manchester, 2020), ch. 5, ‘Primary school teachers, gender and concepts of childhood’.

67 NAGC, *Explorers unlimited*, Spring 1979, ‘Help your fellow man?’, p. 1.

68 Ibid.

69 Ibid.

70 Ibid.

71 NAGC, *Looking to their future*, Apr. 1978, Mrs C. C. Preston, ‘The Esperanto class’, p. 8; NAGC, *Looking to their future*, Nov. 1980, ‘Goings on in Northern Ireland’, p. 12.

72 NAGC, *Dialogue*, Easter 1982, ‘Project Trident: year between scheme’, p. 6.

73 Ibid.

74 NAGC, *Dialogue*, Easter 1982, ‘Last words – first’, p. 3.

75 Susannah Wright, ‘Creating liberal-internationalist world citizens: League of Nations Union junior branches in English secondary schools, 1919–1939’, *Paedagogica Historica*, 56 (2020), pp. 321–40.

76 See, for example, Kenny, ‘Understanding post-war youth culture through oral history’, pp. 233–59.

77 Edwards, ‘“Nothing gets her goat!”‘, p. 27.

78 Kathryn Gleadle and Ryan Hanley, ‘Children against slavery: juvenile agency and the sugar boycotts in Britain’, *Transactions of the Royal Historical Society*, 30 (2020), pp. 97–117; Miller, ‘Assent as agency’; Abebe, ‘Reconceptualising children’s agency’.

79 NAGC, *Looking to their future*, Nov. 1978, ‘The annual general meeting’, p. 20.

80 Ibid., p. 20.

81 Gleadle and Hanley, ‘Children against slavery’, pp. 97–117.

82 NAGC, *Looking to their future*, June 1976, ‘Practical support from the Inner London Education Authority’, p. 1.

83 Judith M. Bennett and Shannon McSheffrey, ‘Early, erotic and alien: women dressed as men in late medieval London’, *History Workshop Journal*, 77 (2014), pp. 1–25; Rachel E. Moss, ‘Ready to disport with you: homosocial culture amongst the wool merchants of fifteenth-century Calais’, *History Workshop Journal*, 86 (2018), pp. 1–21.

84 NAGC, *Looking to their future*, Apr. 1978, ‘A London member looks back’, p. 10; NAGC, *Looking to their future*, June 1976, ‘Practical support from the Inner London Education Authority’, p. 1.

85 NAGC, *Looking to their future*, June 1976, ‘Practical support from the Inner London Education Authority’, p. 1.

86 Bod., Per 264505 e. 4, National Association for Gifted Children, *Journal of the Gifted Child*, Autumn 1979, ‘Saturday Club’, pp. 46–51.

87 Ibid., p. 46.

88 Ibid., p. 46.

89 Ibid., p. 50.

90 Ibid., p. 50.

91 Ibid., p. 51.

92 Ibid., p. 51.

93 Ibid., p. 51.

94 NAGC, newsletter, May 1969, ‘Wolverhampton’, p. 5.

95 Ibid., p. 8.

96 Ibid., p. 8.

97 Ibid., p. 8.

98 NAGC, *Explorers unlimited*, newsletter, Summer 1981, ‘Simon starts school’, p. 28; Bod., Per 264505 e. 4, National Association for Gifted Children, *Journal of the Gifted Child*, Autumn 1979, ‘Helping a gifted child with mathematics’, Yael Naim Dowker and Clifford Hugh Dowker, p. 60; Tisdall, *A progressive education?*, p. 196.

99 NAGC, *Looking to their future*, Nov. 1980, ‘Community spirit?’, p. 29.

100 NAGC, *Dialogue*, Easter 1982, ‘Political forum’, p. 9.

101 NAGC, *Explorers unlimited*, Autumn 1979, ‘Pantophobia: fear of everything’, p. 14.

102 NAGC, *Explorers unlimited* newsletter, Summer 1979, ‘What’s it like then?’, p. 1.

103 Ibid., pp. 1–2.

104 NAGC, *Explorers unlimited* newsletter, Mar. 1981, ‘The tube train’, p. 23; NAGC, *Explorers unlimited* newsletter, Mar. 1981, ‘The Christmas fair’, p. 22.

105 Bod., Per 264505 e. 4, National Association for Gifted Children, *Journal of the Gifted Child*, Autumn 1979, ‘Saturday Club’, p. 46.

106 Ibid., p. 46.

107 NAGC, *Looking to their future*, Autumn 1992, Freida Painter, ‘“High-flyers”– careers in industry’, pp. 13–14.

108 NAGC, *Looking to their future*, May 1977, ‘Country holidays for young explorers’, p. 5.

109 Ibid., p. 5.

110 NAGC, *Looking to their future*, Nov. 1980, ‘Residential courses 1980 or what explorers had to say’, p. 10.

111 Tisdall, *A progressive education?*, pp. 10–12, 52, 72–7.

112 Gleadle and Hanley, ‘Children against slavery’; Miller, ‘Assent as agency’; Abebe, ‘Reconceptualising children’s agency’.

113 Robinson, Schofield, Sutcliffe-Braithwaite, and Thomlinson, ‘Telling stories about post-war Britain’, p. 303.

114 Hughes, ‘Young socialist men in 1960s Britain’; idem, *Young lives on the left*; Cohen, *Folk devils and moral panics*; Osgerby, ‘The good, the bad and the ugly’; Bartie, ‘Moral panics and Glasgow gangs’; Jackson and Bartie, *Policing youth: Britain*.

115 Mills, ‘The experience and memory of youth in England’, pp. 2, 6.

116 For a fantastic recent snapshot of such work, see Pooley and Taylor, *Children*’*s experiences of welfare in modern Britain*.

117 Heywood, ‘On the margins or in the mainstream?’, p. 135; Hendrick interviewed in Smith and Greene, eds., *Key thinkers in childhood studies*, p. 121; Maza, ‘The kids aren’t all right’.

118 Gleason, ‘Avoiding the agency trap’; Thomas, ‘Historicising agency’; Miller, ‘Assent as agency’; Abebe, ‘Reconceptualising children’s agency’.

119 Gleadle and Hanley, ‘Children against slavery’.

120 Davies, Freeman, and Pemberton, ‘“Everyman a capitalist” or “free to choose”‘, p. 500.

